# Studies on the Regulation of (p)ppGpp Metabolism and Its Perturbation Through the Over-Expression of Nudix Hydrolases in *Escherichia coli*

**DOI:** 10.3389/fmicb.2020.562804

**Published:** 2020-10-15

**Authors:** Rajeshree Sanyal, Allada Vimala, Rajendran Harinarayanan

**Affiliations:** Laboratory of Bacterial Genetics, Centre for DNA Fingerprinting and Diagnostics, Hyderabad, India

**Keywords:** (p)ppGpp, stringent response, SpoT, RelA, nudix hydrolases

## Abstract

Stringent response mediated by modified guanosine nucleotides is conserved across bacteria and is regulated through the Rel/Spo functions. In *Escherichia coli*, RelA and SpoT proteins synthesize the modified nucleotides ppGpp and pppGpp, together referred to as (p)ppGpp. SpoT is also the primary (p)ppGpp hydrolase. In this study, using hypomorphic *relA* alleles, we provide experimental evidence for SpoT-mediated negative regulation of the amplification of RelA-dependent stringent response. We investigated the kinetics of ppGpp degradation in cells recovering from stringent response in the complete absence of SpoT function. We found that, although greatly diminished, there was slow ppGpp degradation and growth resumption after a lag period, concomitant with decrease in ppGpp pool. We present evidence for reduction in the ppGpp degradation rate following an increase in pppGpp pool, during recovery from stringent response. From a genetic screen, the nudix hydrolases MutT and NudG were identified as over-expression suppressors of the growth defect of Δ*spoT* and Δ*spoT* Δ*gppA* strains. The effect of over-expression of these hydrolases on the stringent response to amino acid starvation and basal (p)ppGpp pool was studied. Over-expression of each hydrolase reduced the strength of the stringent response to amino acid starvation, and additionally, perturbed the ratio of ppGpp to pppGpp in strains with reduced SpoT hydrolase activity. In these strains that do not accumulate pppGpp during amino acid starvation, the expression of NudG or MutT supported pppGpp accumulation. This lends support to the idea that a reduction in the SpoT hydrolase activity is sufficient to cause the loss of pppGpp accumulation and therefore the phenomenon is independent of hydrolases that target pppGpp, such as GppA.

## Introduction

Stringent response is a stress response ubiquitously found in microorganisms. It is characterized by the accumulation of the signaling molecules ppGpp and pppGpp that are synthesized by the transfer of a pyrophosphate moiety from ATP to GDP or GTP, respectively, and collectively referred to as (p)ppGpp (Cashel et al., 1996). Accumulation of (p)ppGpp modifies the cellular physiology globally resulting in the cell switching from a growth and proliferation mode to a survival mode (Chatterji and Kumar Ojha, 2001; Braeken et al., 2006; Potrykus and Cashel, 2008; Hauryliuk et al., 2015). In the gram-negative model organism *Escherichia coli*, nutritional starvation signals activate the stringent response leading to the accumulation of (p)ppGpp and reprogramming of transcription (Durfee et al., 2008; Traxler et al., 2011). This is brought about by the binding of (p)ppGpp to RNA polymerase that is facilitated by DksA, an RNA polymerase binding protein (Ross et al., 2013, 2016; Zuo et al., 2013). Additionally, (p)ppGpp binds several proteins and alters their activity (Wang et al., 2019). (p)ppGpp is required for bacterial virulence, and in many pathogens, the expression and activity of virulence regulators are integrated into a global response mediated by ppGpp, thereby coupling pathogenesis to metabolic status (Dalebroux et al., 2010).

In β- and γ-proteobacteria, including *E. coli*, (p)ppGpp metabolism is primarily driven by the paralogs, RelA and SpoT, which are members of the multi-domain Rel/Spo homolog (RSH) family of proteins (Mittenhuber, 2001; Atkinson et al., 2011). The arrangement of domains within the RSH proteins are conserved. The domains responsible for (p)ppGpp synthesis and hydrolysis are carried within the N-terminal half of the protein, while domains implicated in regulatory functions reside within the C-terminal half of the protein. The (p)ppGpp synthetase and hydrolase domains are functional in SpoT, while the latter domain is non-functional in RelA due to the presence of mutations. The data available in the literature suggest that the stress signals are sensed by the regulatory domains of the RSH proteins, leading to conformation changes that modulate the synthase or hydrolase activity. RelA is a ribosome-bound protein activated by the “hungry” codons that appear following amino acid starvation and the consequent increase in the concentration of uncharged tRNA (Cashel et al., 1996). Cryo-electron microscopy studies have provided insights on the structural basis for RelA activation by the entry of uncharged tRNA into the A-site of a translating ribosome and as well as the role of C-terminal domains in ribosome interaction (Arenz et al., 2016; Brown et al., 2016; Loveland et al., 2016). A recent report has presented evidence for accumulation of uncharged tRNA^*Lys*^ in response to fatty acid starvation and consequent activation of RelA-dependent (p)ppGpp synthesis (Sinha et al., 2019).

In the absence of stress, SpoT is associated with a weak synthase and a strong (p)ppGpp hydrolase activity, and the hydrolase function is essential for the growth of *E. coli* (An et al., 1979; Xiao et al., 1991). The basal (p)ppGpp pool in *E. coli* is regulated through SpoT activity (Sarubbi et al., 1988) and it can elicit stringent response following carbon (Xiao et al., 1991), fatty acid (Seyfzadeh et al., 1993), and iron (Vinella et al., 2005) limitation. The SpoT hydrolase activity was inhibited by uncharged tRNA and the inhibition was more severe in the presence of ribosomes (Richter, 1980), conditions that mimic amino acid starvation. Interaction of SpoT with other factors regulate the balance between its synthase and hydrolase functions. It has been reported that the acyl carrier protein interacts with the TGS domain of SpoT and increases the synthase activity during fatty acid starvation (Battesti and Bouveret, 2006). During exponential growth the interaction of SpoT with the GTPase, CgtA/ObgE can modulate the hydrolase activity during exponential growth (Jiang et al., 2007). In a recent report, a small protein called YtfK was proposed to activate stringent response by tilting the catalytic balance of SpoT toward (p)ppGpp synthesis (Germain et al., 2019). In addition to SpoT, pppGpp is hydrolyzed into ppGpp by the guanosine pentaphosphate phosphohydrolase GppA in *E. coli* (Somerville and Ahmed, 1979; Harat and Sy, 1983; Keasling et al., 1993). The physiological significance of the conversion of pppGpp to ppGpp is not apparent. However, pppGpp was found to be less potent than ppGpp with respect to regulation of growth rate, RNA/DNA ratios, ribosomal RNA P1 promoter transcription inhibition, threonine operon promoter activation and RpoS induction in *E. coli* (Mechold et al., 2013). When pppGpp hydrolysis was prevented by the elimination of SpoT and GppA functions, RelA mediated (p)ppGpp synthesis was activated in the absence of starvation (Sanyal and Harinarayanan, 2020). Many (p)ppGpp binding and metabolizing proteins were identified from a DRaCALA (differential radial capillary action of ligand assay) based screen, but interestingly, both SpoT and GppA were not identified in this screen (Zhang et al., 2018). One way to examine the role of hydrolases other than SpoT in the turn-over of (p)ppGpp can be through studying a Δ*spoT* strain. Although SpoT hydrolase activity is essential for the growth of wild type *E. coli*, non-inactivating suppressor mutations in the *relA* locus have been found to rescue the growth defect of Δ*spoT* strain (Montero et al., 2014; Sanyal and Harinarayanan, 2020). These *relA* hypomorphs can be used to address (p)ppGpp turnover in the absence SpoT function.

In this study, using hypomorphic *relA* alleles or the over-expression of nudix hydrolases – conditions that support *E. coli* growth in the absence of SpoT function, we have examined the role of SpoT hydrolase function in (p)ppGpp metabolism during RelA mediated stringent response to amino acid starvation. Specifically, we have studied, (i) the role of SpoT hydrolase activity in the regulation of the amplification of the *relA*-mediated stringent response after amino acid starvation; (ii) the degradation of stringent nucleotide ppGpp in the absence of SpoT activity; (iii) the effect of increase in pppGpp pool on the SpoT mediated turnover of (p)ppGpp, and (iv) the effect of over-expression of nudix hydrolases, NudG and MutT, on the (p)ppGpp pool during stringent response in wild type strain and strains with lowered SpoT hydrolase activity. Our results indicate, the SpoT hydrolase activity modulates the quorum of deacylated tRNA required for RelA dependent increase in (p)ppGpp. Our results also show that the cellular pppGpp pool responds to the overall (p)ppGpp hydrolase activity of the cell in a counterintuitive manner, that is, when the cellular hydrolase activity was lowered its pool size decreased and when the hydrolase activity was increased using nudix hydrolases, its pool size increased.

## Results

### Growth Phenotype of Strains Carrying Hypomorphic *relA* Alleles in SMG

**FIGURE 1:**
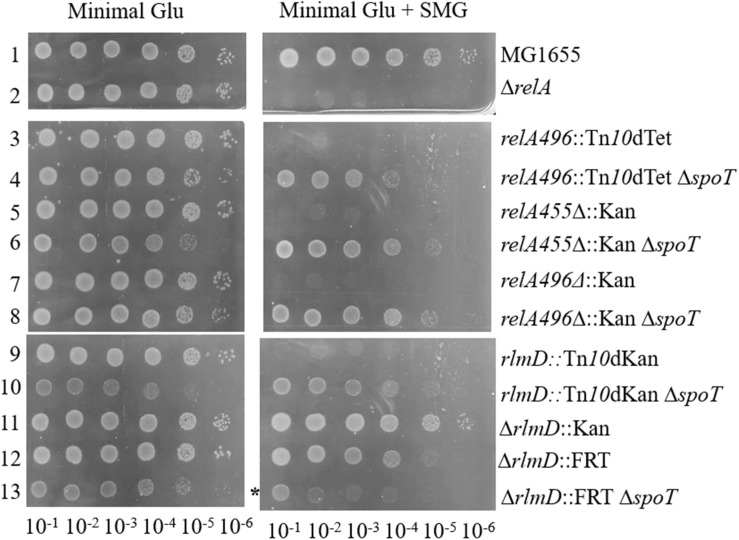
SMG resistance phenotype of hypomorphic RelA alleles was modulated by SpoT function. Cultures grown to saturation were washed, serially diluted, and spotted on minimal medium containing glucose with or without SMG (serine, methionine, and glycine) and photographed after 20 h incubation at 37°C. The relevant strain genotypes are indicated. *Growth in the presence of SMG was significantly retarded but not abolished. The strains from rows 1 to 13 are – RS1, RS8, RS9, RS17 white colony, RS53, RS92 white colony, RS54, RS420 white colony, RS11, RS18 white colony, RS303, RS316, RS361 white colony. Strains and their genotype are listed in Supplementary Table 1. The white colony of a strain refers to the derivative cured of plasmid P_*lac*_-spoT^+^.

**FIGURE 2:**
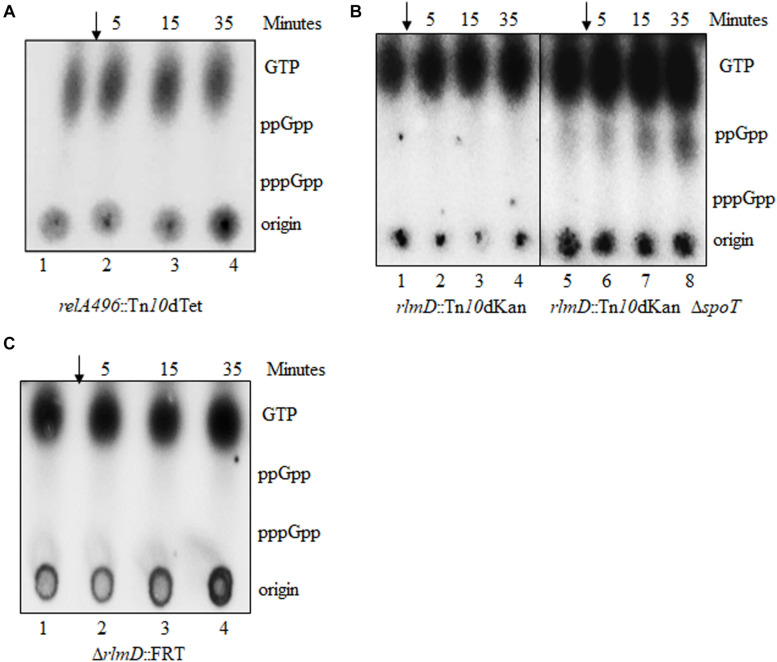
Hypomorphic *relA* alleles do not accumulate (p)ppGpp in response to isoleucine starvation. (p)ppGpp accumulation in response to isoleucine starvation was monitored in strains carrying the following *relA* alleles *relA496*:Tn*10*dTet **(A)**, *rlmD*:Tn*10*dKan and *rlmD*:Tn*10*dKan Δ*spoT* **(B)**, and Δ*rlmD*:FRT **(C)**. Strains were cultured in MOPS glucose medium, labeled with P^32^, isoleucine starvation was induced with valine (arrow), and samples were collected immediately before valine addition and subsequently at the times indicated above the lanes and subjected to PEI-TLC (see section “Materials and Methods” for details). Data presented is a representative of experiments done at least 3 times. Strains used are RS9 **(A)**, RS11 and RS18 white colony **(B)**, and RS316 **(C)**. The white colony of a strain refers to the derivative cured of plasmid P_*lac*_-spoT^+^.

We had reported the isolation of two hypomorphic *relA* alleles, namely *relA*:Tn*10*dTet and *rlmD*:Tn*10*dKan through transposon mutagenesis that suppressed the growth defect of the Δ*spoT* strain (Sanyal and Harinarayanan, 2020). We expected the *relA*:Tn*10*dTet transposon insertion after the 496^*th*^ codon in the *relA* ORF to lead to synthesis of truncated RelA polypeptide (full length RelA is 744 amino acids), therefore, early stop codons were introduced in the *relA* ORF (see methods) to allow synthesis of truncated RelA polypeptides with the N-terminal 455 amino acids (*relA455*Δ:Kan) or 496 amino acids (*relA496*Δ:Kan). *relA* allele encoding the N-terminal 455 aa of RelA polypeptide has been expressed from plasmid, the truncated protein synthesized (p)ppGpp constitutively and did not respond to amino acid starvation (Schreiber et al., 1991; Svitil et al., 1993). The phenotype of these alleles was qualitatively compared with the *relA*:Tn*10*dTet transposon insertion by studying growth on medium containing the amino acids serine, methionine, and glycine (SMG), which is known to induce isoleucine starvation (Uzan and Danchin, 1976). The *relA* null mutant or an allele with very low activity such as *relA1* (Metzger et al., 1989) does not grow in this medium (Figure 1, rows 1 and 2). Strains carrying the *relA*:Tn*10*dTet, *relA455*Δ:Kan, or *relA496*Δ:Kan allele exhibited a similar SMG-sensitive phenotype (Figure 1, rows 3, 5, and 7). However, the growth sensitivity was overcome after the elimination of SpoT function (Figure 2, rows 4, 6, and 8). These results suggest, after isoleucine starvation, the truncated RelA polypeptides, unlike full length RelA, are unable to increase the (p)ppGpp pool due to the presence of SpoT hydrolase activity. Notably, over-expression of truncated RelA polypeptide from plasmid confers SMG resistance (data not shown) and causes gratuitous stringent response. As expected for a RelA polypeptide lacking the C-terminal domain necessary for interaction with the ribosome, the *relA*:Tn*10*dTet allele did not respond to amino acid starvation (Figure 2A). Stringent response elicited in the presence of SMG or after amino acid starvation by the *relA* alleles studied here has been tabulated (Table 1). The above results highlight the importance of SpoT hydrolase activity in regulating stringent response from RelA variants incapable of ribosome interaction. This may be useful to understand the stringent response elicited through small alarmone synthases.

**TABLE 1:** SMG and amino acid starvation phenotypes of *relA* alleles.

Genotype	SMG phenotype^a^	Amino acid starvation^b^
Wild type	R	ppGpp and pppGpp
Δ*relA*	S	None
*relA496*:Tn10dTet	S	None
*relA496*:Tn10dTet Δ*spoT*	R	None
*relA455*Δ:Kan	S	None
*relA455*Δ:Kan Δ*spoT*	R	None
*relA496*Δ:Kan	S	None
*relA496*Δ:Kan Δ*spoT*	R	None
*rlmD*:Tn10dKan	S	None
*rlmD*:Tn10dKan Δ*spoT*	R	None
Δ*rlmD*:Kan	R	ND^*c*^
Δ*rlmD*:FRT	R	None
Δ*rlmD:FRT* Δ*spoT*	R	ppGpp

*^*a*^Growth in the presence of serine, methionine, and glycine; R- resistant, S- sensitive; ^*b*^stringent nucleotides in response to amino acid starvation; ^*c*^not determined.*

**FIGURE 3:**
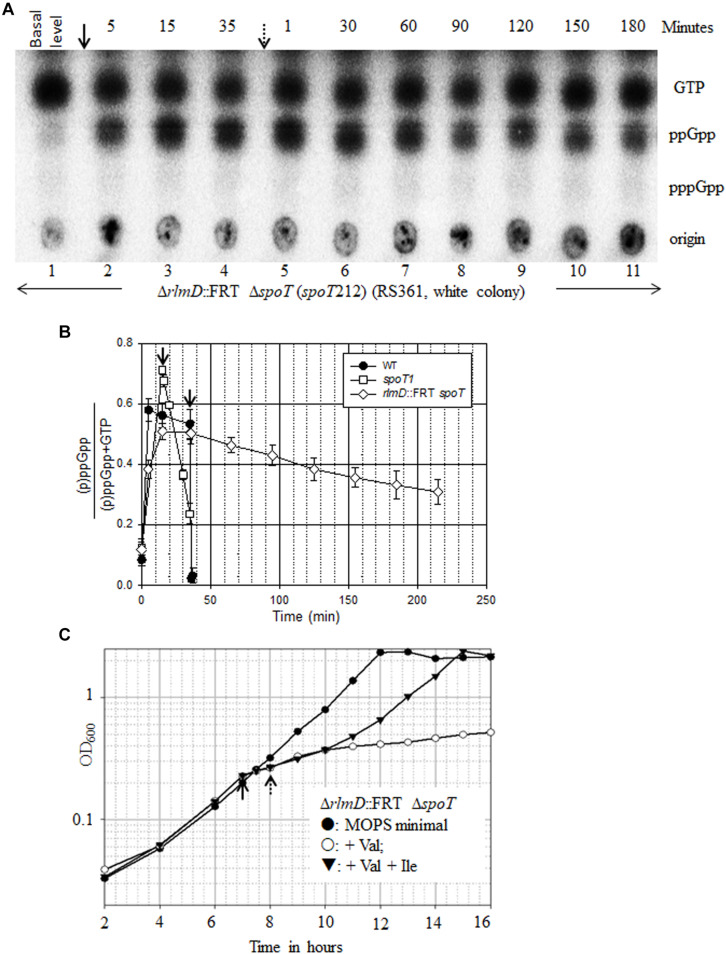
ppGpp turnover in the absence of SpoT function and its effect on growth. **(A)** The Δ*rlmD*:FRT Δ*spoT* strain (RS361, white colony) was cultured in MOPS minimal medium containing glucose, labeled with P^32^ and subjected to PEI-TLC. Isoleucine starvation was induced with valine (arrow), and samples were collected immediately before valine addition and subsequently at time points indicated above the lanes. Starvation was reversed by the addition of isoleucine (dotted arrow), and samples were collected at the time points indicated. The white colony of a strain refers to the derivative cured of plasmid P_*lac*_-spoT^+^. Data presented is representative of experiments done 3 times. **(B)** The amount of (p)ppGpp over total [(p)ppGpp + GTP] at different time points after isoleucine starvation and after the reversal of starvation was plotted for the strains indicated using the data in Supplementary Tables 2, 3, 5. **(C)** Δ*rlmD*:FRT Δ*spoT* strain was grown in MOPS minimal medium containing glucose and subjected to isoleucine starvation by the addition of valine (solid arrow) and subsequently reversed by the addition of isoleucine (dotted arrow). Data from a representative experiment was plotted.

The *rlmD*:Tn*10*dKan transposon insertion was very close to the 3′-end of *rlmD*, the gene immediately upstream of RelA (Sanyal and Harinarayanan, 2020). Therefore, it was expected that the *relA* expression could be lowered in this strain due to premature termination of transcripts initiating from the promoters upstream of the insertion. To test the role of the promoters within the *rlmD* ORF (Metzger et al., 1988; Nakagawa et al., 2006; Brown et al., 2014) in the SMG resistance phenotype, the Δ*rlmD*:kan allele from Keio collection (Baba et al., 2006) and Δ*rlmD*:FRT derivative obtained by FLP mediated excision of the Kan^*R*^ determinant (Datsenko and Wanner, 2000) were tested for stringent response using the SMG test. While the *rlmD*:Tn*10*dKan strain did not grow in the SMG plate in the presence of SpoT function (Figure 1, rows 9 and 10), this was not the case for strains carrying the Δ*rlmD*:kan or Δ*rlmD*:FRT alleles. Growth of the Δ*rlmD*:FRT strain was significantly slower in the presence of Δ*spoT* allele, irrespective of the presence or absence of SMG. This may be due to increase in the basal (p)ppGpp pool, which is known to reduce the growth rate (Sarubbi et al., 1988, 1989). The Δ*rlmD*:kan Δ*spoT* strain was lethal (data not shown). Isoleucine starvation did not elicit stringent response in the *rlmD*:Tn*10*dKan, Δ*rlmD*:FRT, and *rlmD*:Tn*10*dKan Δ*spoT* strains (Figures 2B,C), but importantly, stringent response was elicited in the Δ*rlmD*:FRT Δ*spoT* strain (Figure 3A, lanes 1–4). This indicated, the hydrolase activity of SpoT prevented the accumulation of (p)ppGpp following amino acid starvation in the case of Δ*rlmD*:FRT allele. The *rlmD*:Tn*10*dKan allele failed to accumulate (p)ppGpp even in the absence of SpoT hydrolase activity, however, the removal of SpoT function conferred SMG-resistance in this strain (Figure 1 row 10) and suggested that there was an increase in the basal (p)ppGpp concentration.

The above data is consistent with an idea that there can be at least two levels of negative regulation of (p)ppGpp accumulation during RelA-mediated stringent response. One is the SpoT mediated degradation of (p)ppGpp, which is illustrated by the accumulation of (p)ppGpp in the Δ*rlmD*:FRT strain only after the loss of SpoT function (compare Figures 2C, 3A). A second SpoT-independent regulation may explain the absence of (p)ppGpp accumulation in the *rlmD*:Tn*10*dKan Δ*spoT* strain, wherein, hydrolases other than SpoT may be involved in the turnover of the small amounts of (p)ppGpp synthesized. The small amount of ppGpp observed in the *rlmD*:Tn10dKan Δ*spoT* strain (as compared to the isogenic *rlmD*:Tn10dKan strain) can be attributed to over-loading of TLC as seen from the higher intensity of the GTP spots.

### ppGpp Degradation and Growth Recovery From Stringent Response in the Absence of SpoT Function

**FIGURE 4:**
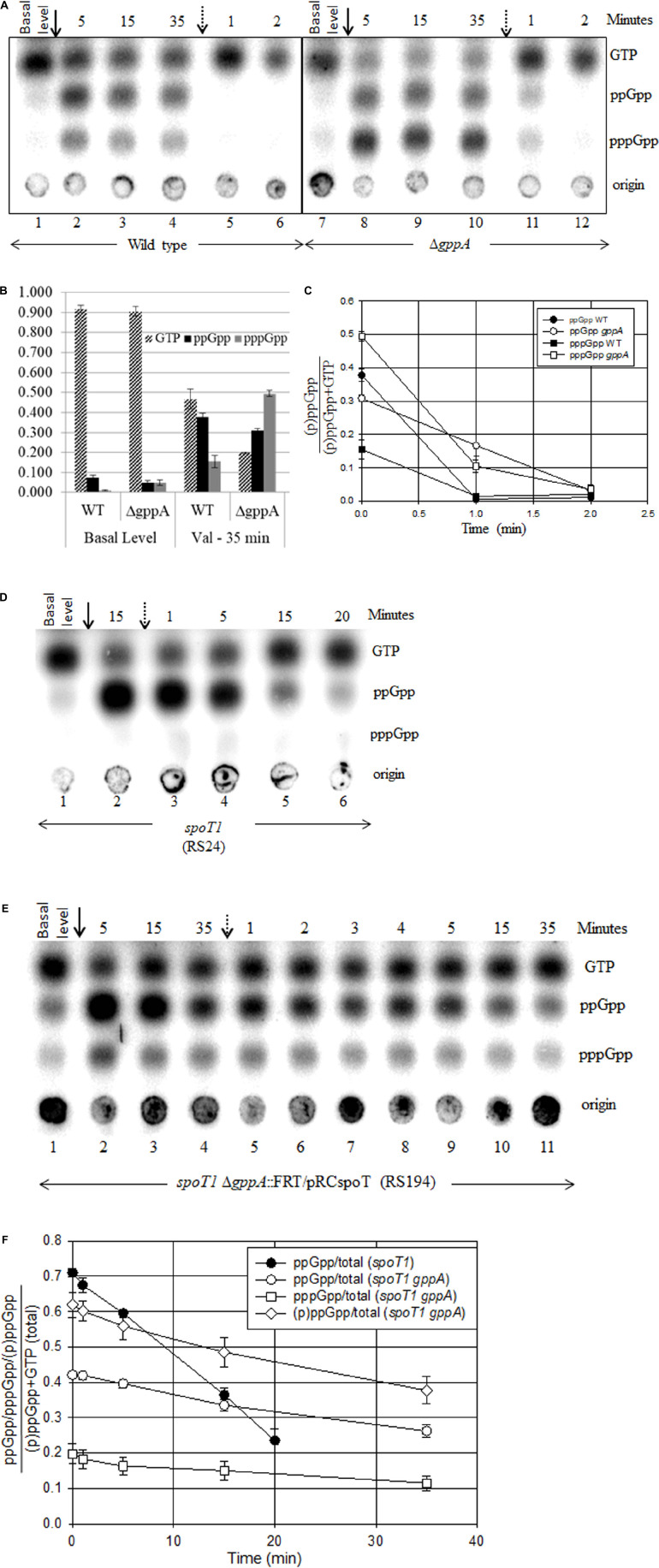
Effect of SpoT and/or GppA hydrolase activity on the synthesis and turnover of stringent nucleotides during amino acid starvation and recovery. Isoleucine starvation was induced by the addition of valine (solid arrow) and reversed by the addition of isoleucine (dotted arrow) in the wild type and Δ*gppA* **(A)**, *spoT1* **(D)**, and *spoT1* Δ*gppA*/P_*lac*_-spoT^+^ strain after reducing the *spoT* expression by allowing growth in the absence of IPTG **(E)**. A representative TLC is shown for each strain. **(B)** The concentration of ppGpp, pppGpp, or GTP over total [(p)ppGpp + GTP] at the time points indicated was plotted as bar graph using data from two independent experiments (Supplementary Table 5). Fraction of ppGpp or pppGpp or (p)ppGpp over total [(p)ppGpp + GTP] after the reversal of starvation was plotted for the wild type and *gppA* mutant with data from two independent experiments **(C)** the *spoT1* and *spoT1* Δ*gppA*/P_*lac*_-spoT^+^ strains **(F)** with data from three independent experiments (Supplementary Tables 3, 4). The strains are wild type (RS1), Δ*gppA* (RS307 white colony), *spoT1* (RS24), and *spoT1* Δ*gppA*:FRT/P_*lac*_-spoT^+^ (RS194). The white colony of a strain refers to the derivative cured of plasmid P_*lac*_-spoT^+^.

When subjected to isoleucine starvation, the Δ*rlmD*:FRT Δ*spoT* strain accumulated ppGpp (Figure 3A). Strikingly, there was no accumulation of pppGpp unlike in a wild type strain. This has been reported for the hydrolase deficient *spoT1* allele and was called the “spotless” phenotype (Laffler and Gallant, 1974). The *spoT1* encoded protein has three changes, a substitution (H255Y) and a two amino acid insertion between residues 82 and 83 (+QD) as compared to the *spoT*^+^ encoded protein (Spira et al., 2008). We studied the synthesis and turnover rate of ppGpp in the complete absence of SpoT function using this strain. Following valine induced isoleucine starvation, the rate of ppGpp accumulation was diminished, with the ppGpp level continuing to increase beyond 5 min unlike in the wild type strain (Figures 3A,B, 4A). This may be attributed to lowered *relA* expression in the Δ*rlmD*:FRT genetic background. After the reversal of amino acid starvation by isoleucine addition, the rate of ppGpp degradation was greatly diminished (Figure 3A). There was 38% decrease in ppGpp pool 180 min after the reversal of starvation (Figure 3B and Supplementary Table 2). The degradation rates are lower than that seen in the hydrolase defective *spoT1* strain, where there was 67% decrease in ppGpp pool 20 min after the reversal of starvation (Figures 3B, 4D and Supplementary Table 3). These results indicate SpoT as the primary ppGpp hydrolase during stringent response. However, the very slow turnover of (p)ppGpp in the Δ*spoT* background suggest to the possible existence of alternative hydrolases (see below).

Since an increase in (p)ppGpp pool inhibits growth rate (Sarubbi et al., 1988; Sanyal and Harinarayanan, 2020), we studied how the slower ppGpp accumulation and reduced ppGpp degradation in the Δ*rlmD*:FRT Δ*spoT* strain affected growth during onset and reversal of isoleucine starvation. For comparison, we also studied the kinetics of growth arrest and recovery in the wild type, *spoT1*, and Δ*rlmD*:FRT strains. As expected, growth ceased following the addition of valine and resumed after the addition of isoleucine in the wild type strain (Supplementary Figure 1A). In the *spoT1* strain, the kinetics of isoleucine starvation-induced growth arrest was similar to that observed the wild type, but there was a lag of ∼30 min before growth resumed after the reversal of starvation (Supplementary Figure 1B). This is consistent with the reduced rate of ppGpp degradation in the *spoT1* strain (Figure 4D and Supplementary Table 3). Growth arrest after isoleucine starvation in the Δ*rlmD*:FRT Δ*spoT* strain was similar to that in the wild type and *spoT1* strains, but following the reversal of starvation, growth resumed after a lag of 120 min, when the ppGpp pool was 54% of GTP (Figure 3C and Supplementary Table 2). One hour after growth resumed, that is, 180 min after isoleucine addition, the ppGpp pool was 45% of GTP. Our data indicates, ppGpp > 54% of GTP conferred growth inhibition as strong as that observed during amino acid starvation. There was gradual growth recovery as the ppGpp pool dropped, with the growth rate inversely proportional to ppGpp concentration as reported (Sarubbi et al., 1988). Unlike the Δ*rlmD*:FRT Δ*spoT* strain, growth resumed immediately after the reversal of isoleucine starvation in the isogenic Δ*rlmD*:FRT strain, which did not accumulate ppGpp (Supplementary Figure 1C and Figure 2C). This indicated, the reduced turnover of ppGpp was the sole cause of growth lag in the former strain.

Previously, we had reported RelA-dependent synthetic lethality in the *spoT1* Δ*gppA* strain with an associated accumulation of (p)ppGpp (Sanyal and Harinarayanan, 2020). Consistent with this, we observed growth inhibition in the *spoT1* Δ*gppA*/P_*lac*_-spoT^+^ strain after lowering *spoT* gene expression by IPTG withdrawal (Supplementary Figure 2). We compared the level of (p)ppGpp or ppGpp as a ratio of GTP in the *spoT1* Δ*gppA*/P_*lac*_-spoT^+^ and Δ*rlmD*:FRT Δ*spoT* strains, respectively, to the extent of growth inhibition. Accumulation of ppGpp to 45% of GTP in the Δ*rlmD*:FRT Δ*spoT* strain, 180 min after addition of isoleucine (Supplementary Table 2) increased the doubling time 1.7-fold from 90 to 156 min (Figure 3C). On the other hand, accumulation of ppGpp and pppGpp to 38 and 14% of GTP, respectively, in the *spoT1* Δ*gppA*/P_*lac*_-spoT^+^ strain after IPTG withdrawal (Figure 4E lane 1 and Supplementary Table 4) conferred a significantly stronger growth inhibition – 4.9-fold increase in doubling time from 78 to 384 min (Supplementary Figure 2). Assuming, the growth properties of the strains were primarily determined by the intracellular pool of stringent nucleotides, we attribute the stronger growth inhibition in the latter strain to the presence of pppGpp.

### Increase in pppGpp Reduced the Degradation Rate of ppGpp

As reported, only ppGpp accumulation was observed after amino acid starvation in the hydrolase deficient *spoT1* mutant, and the degradation rate of ppGpp was significantly reduced during recovery from stringent response (Figure 4D; Laffler and Gallant, 1974). We wanted to test if the SpoT hydrolase defect would also affect the degradation of pppGpp and if the presence of pppGpp would perturb ppGpp degradation. In the *spoT1* Δ*gppA*/P_*lac*_-spoT^+^ strain, isoleucine starvation after lowering *spoT* gene expression caused further accumulation of ppGpp and pppGpp (Figure 4E). We studied the degradation rate of the stringent nucleotides during recovery from isoleucine starvation in this strain and compared it with that seen in the g*ppA* and *spoT1* strains.

Consistent with GppA being a pppGpp hydrolase, the pppGpp pool increased relative to ppGpp in the Δ*gppA* strain (Figure 4A). However, in the SpoT hydrolase deficient background, the pppGpp pool was significantly lower than ppGpp, despite the absence of GppA activity (Figure 4E). In the *gppA* mutant, the GTP and pppGpp pools were, respectively, 2.5-fold lower and 3.8-fold higher as compared to the wild type strain, while the ppGpp pool did not change significantly (Figures 4A,B and Supplementary Table 5). While a relative increase in the pppGpp pool was expected in the *gppA* mutant, the decrease in GTP pool is not consistent with current knowledge. Degradation of pppGpp to GTP is primarily SpoT hydrolase mediated and there is no evidence to our knowledge this can be inhibited in a *gppA* mutant. As compared to the wild type, in the Δ*gppA* strain, after the inhibition of RelA activity by isoleucine addition, a significant decrease in the degradation rate of ppGpp was observed (Figures 4A,C and Supplementary Table 5). In the *spoT1* Δ*gppA*:FRT/P_*lac*_-spoT^+^ strain recovering from isoleucine starvation, there was 22 and 19% degradation of ppGpp and pppGpp, respectively, 15 min after the reversal of isoleucine starvation (Figures 4E,F and Supplementary Table 4). The degradation rate of ppGpp was more than two-fold lower than in the *spoT1* strain, where 54% of ppGpp was degraded 15 min after reversal of starvation (Figure 4F and Supplementary Table 3). The results show that, increasing the concentration of pppGpp slows down the SpoT-mediated degradation of ppGpp. From the data we have calculated the half-lives of (p)ppGpp using the formula t_1__/__2_ = A_0_/2K for a zero-order reaction, where A_0_ is the initial concentration and K the slope. From less than a minute in the wild-type strain the half-life of ppGpp increases to 15 min in the *spoT1* strain and to 229 min in the Δ*rlmD*:FRT Δ*spoT* strain. In the presence of pppGpp the half-lives of ppGpp and pppGpp in the *spoT1* strain was 47 and 41 min, respectively.

To explain the above results, we would like to propose a model based on differential substrate specificity of the SpoT protein. In an earlier study, by comparing the SpoT mediated hydrolysis of ppGpp and pppGpp separately and when presented together at equimolar concentration *in vitro*, it was shown that SpoT did not exhibit substrate preference (Mechold et al., 1996). To explain our observation, we would like to propose that when pppGpp is present at a higher concentration than ppGpp, it competitively inhibits the hydrolysis of ppGpp by SpoT *in vivo*. However, the hydrolase deficient protein encoded by the *spoT1* allele is more sensitive to the inhibition of ppGpp hydrolysis by pppGpp. Reduced ppGpp hydrolysis can slow down GTP regeneration if the hydrolysis of ppGpp to GDP by SpoT and subsequent conversion of GDP to GTP by nucleotide diphosphate kinase (NDK) is the predominant GTP regeneration pathway during the stringent response.

### Genetic Evidence for Compensation of SpoT Requirement by Over-Expression of MutT or NudG

**FIGURE 5:**
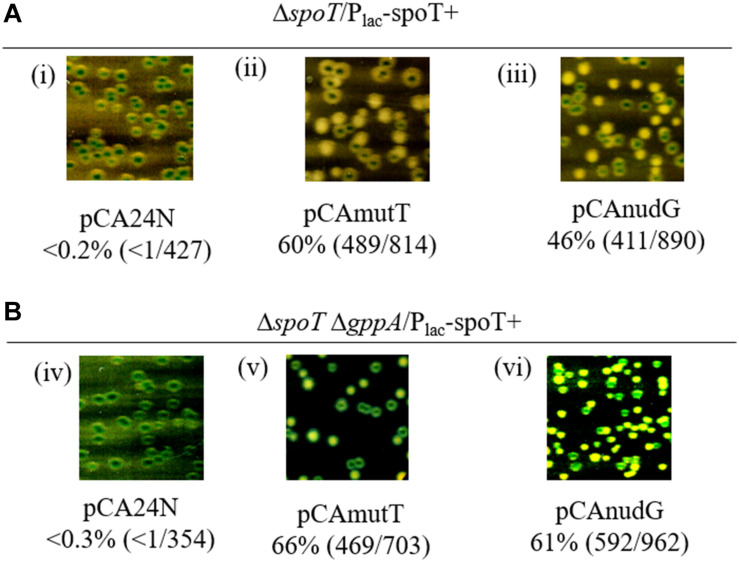
Suppression of SpoT requirement by over-expression of *mutT* or *nudG*. Plasmid segregation assay was used to study the role of *mutT* and *nudG* genes in suppression of the growth defect of Δ*spoT* and Δ*spoT* Δ*gppA* strains in LB medium containing IPTG and Cm. A representative section from the plate has been included for each strain to show the color of the colonies after non-selective growth. The percentage of white colonies and the number of white colonies over the total number of colonies scored (white + blue) are provided for each panel. The ASKA plasmids carrying the *mutT* or *nudG* gene was used and the plasmid vector served as control. The Δ*spoT*/P_*lac*_-spoT^+^ strain was grown in the presence of 0.1 mM IPTG **(A)** and the Δ*spoT* Δ*gppA*/P_*lac*_-spoT^+^ strain was grown in the presence of 1 mM IPTG **(B)**. The strains in panels (i–vi) are RS444, RS680, RS681, RS684, RS685, and RS686, respectively.

Previously, we reported that the growth of Δ*rlmD* Δ*spoT* strain was dependent on GppA function (Sanyal and Harinarayanan, 2020). Using a multi-copy plasmid library of *E. coli* genes, we identified and sequenced plasmid clones that suppressed the growth defect of the Δ*rlmD* Δ*spoT* Δ*gppA* strain (details under section “Materials and Methods”). Among the plasmid clones conferring suppression, many carried the *spoT* or the *gppA* gene (which was expected). Additionally, the *nudG* gene was identified in three independent clones, and it was the only full-length gene in one of the three clones. This suggested, over-expression of *nudG* gene function could be responsible for suppression of growth defect. NudG is a member of the Nudix hydrolase superfamily of proteins and reported to be primarily a 5-hydroxy-CTP diphosphatase (Fujikawa and Kasai, 2002). We also identified a plasmid clone having the *coaE*’-*zapD*-*yacZ*-*mutT-secA*’ genomic fragment that suppressed growth defect. Since this fragment included the *mutT* gene, which, like *nudG*, is a nudix hydrolase, an 8-oxo-dGTP diphosphatase (Akiyama et al., 1987; Bhatnagar and Bessman, 1988), we considered it a candidate gene responsible for suppression. We used the unstable plasmid based segregation assay (described under section “Materials and Methods”) to show that plasmids with chromosomal DNA fragment having *nudG* or the *mutT* gene suppressed the Δ*rlmD*:FRT Δ*spoT* Δ*gppA* synthetic lethality in LB as well as defined media (Supplementary Figure 3). Suppression of Δ*rlmD*:FRT Δ*spoT* Δ*gppA* synthetic lethality by a subset of multi-copy clones and their effect on the growth defect of the Δ*relA* Δ*spoT* strain on minimal medium was studied (Supplementary Table 6). As expected, all clones suppressed the synthetic lethality and only the clone with *spoT* gene supported growth of Δ*relA* Δ*spoT* strain on minimal medium. We used corresponding plasmid clones from the ASKA plasmid collection to ask if the *nudG* or *mutT* gene functions were sufficient to suppress the growth defect arising from the loss of SpoT function and both SpoT and GppA functions. Plasmids pCA24N (vector), pCAmutT, and pCAnudG were individually introduced into the Δ*spoT*/P_*lac*_-spoT^+^ and Δ*spoT* Δ*gppA*/P_*lac*_-spoT^+^ strains to ask if the over-expression of each nudix hydrolase could compensate for the loss of SpoT and as well as the loss of both SpoT and GppA [which accentuates the growth defect from reduction in SpoT hydrolase function (Sanyal and Harinarayanan, 2020)]. Plasmid segregation assay showed that, SpoT function or both SpoT and GppA functions were dispensable in the presence of pCAnudG or pCAmutT plasmid, but not the vector pCA24N (Figure 5). The simplest explanation for these results will be that NudG or MutT over-expression prevents the accumulation of (p)ppGpp. Indeed, it has been shown that NudG and MutT proteins bind and metabolize (p)ppGpp (Zhang et al., 2018). It is interesting that MutT and NudG were identified from two completely different screens. In the study by Zhang et al. (2018), where systematic screening was carried out using DRaCALA (differential radial capillary action of ligand assay) for (p)ppGpp binding proteins, NudG and MutT were identified amongst other proteins. It was shown that these proteins hydrolyzed ppGpp to pGp and this was competed out by the natural nucleotide substrates of the proteins, namely, 8-oxo-(d)GTP (MutT) and 2-OH-(d)ATP (NudG). The ability to delete *spoT* under conditions of MutT or NusG over-expression suggested that they constitute alternative (p)ppGpp degradation pathways.

The hydrolase deficient *spoT202* and *spoT203* alleles elevate the basal ppGpp pool (Sarubbi et al., 1988) and supported the growth of Δ*relA* strain after SMG induced isoleucine starvation (Supplementary Table 7). Introduction of ASKA plasmid carrying the *nudG* or *mutT* gene into the Δ*relA spoT202* and Δ*relA spoT203* strains inhibited the growth of these strains in the presence of SMG, and also improved their growth in the absence of SMG (Supplementary Table 7). This growth pattern is consistent with the lowering of basal (p)ppGpp pool by over-expression of nudix hydrolases.

### Over-Expression of NudG or MutT Alleviates Stringent Response and Restores pppGpp Accumulation in Strains With Reduced SpoT Hydrolase Activity

**FIGURE 6:**
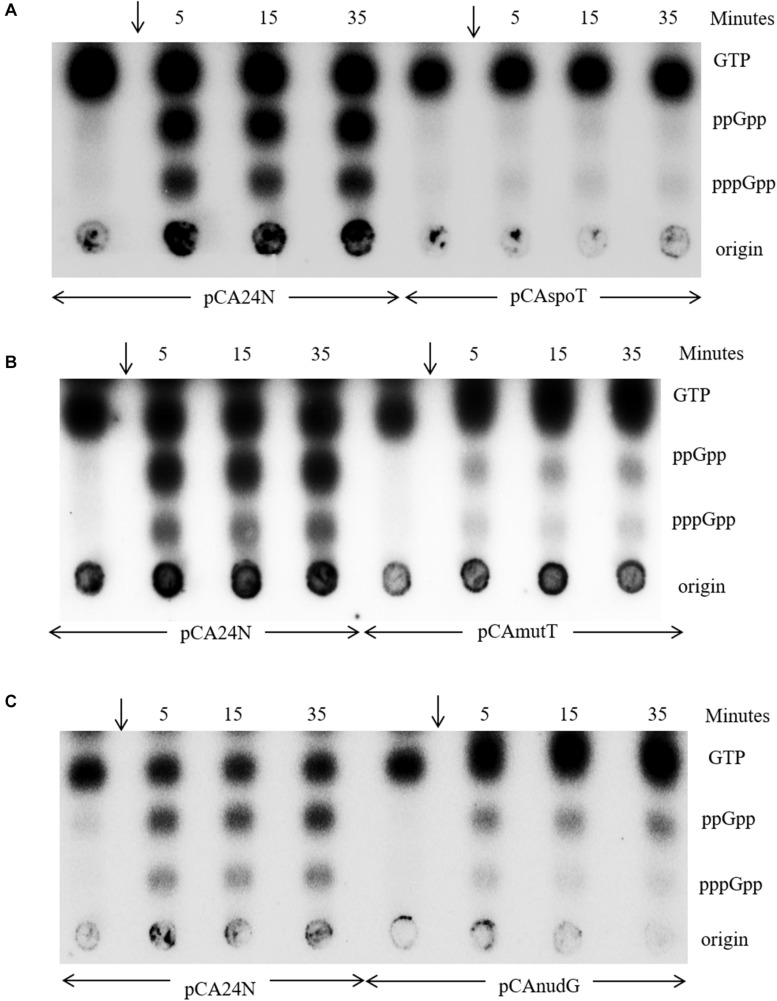
Increased expression of *spoT* or *mutT* or *nudG* alleviates the stringent response to isoleucine starvation. A representative TLC of MG1655 Δ*lacZYAI*:FRT strain carrying the ASKA plasmids indicated below each panel was cultured in MOPS minimal medium containing glucose Cm and 0.1 mM IPTG. The culture was labeled with P^32^ to follow the accumulation of stringent nucleotides after isoleucine starvation by the addition of valine (arrow). Samples were collected immediately before the addition of valine or at the time points indicated and subjected to PEI-TLC. The strains in panels **(A–C)** are RS688, RS760, RS689, and RS690.

Isoleucine starvation in the Δ*rlmD*:FRT strain showed that when the *relA* expression was reduced, physiological levels of SpoT hydrolase activity was sufficient to inhibit stringent response (Figures 2C, 3A). It is therefore possible, with increased expression of SpoT, there could be a proportionate increase in the hydrolase activity so that the stringent response elicited through amino acid starvation could be alleviated in an otherwise wild type strain. To test this, *spoT* expression was induced from plasmid pCAspoT (ASKA collection) (Kitagawa et al., 2005) in the wild type strain and then subjected to isoleucine starvation. While a wild type like stringent response was observed in the presence of plasmid vector, (p)ppGpp accumulation was almost completely abolished after SpoT expression (Figure 6A). This indicated the (p)ppGpp synthesized could be completely hydrolyzed due to net increase in hydrolase activity following over-expression of SpoT. Stringent response to isoleucine starvation was also alleviated during the over-expression of nudix hydrolases NudG or MutT (Figures 6B,C). However, unlike SpoT over-expression, some residual (p)ppGpp accumulation was observed. Although this seems to suggest (p)ppGpp is less efficiently hydrolyzed by the nudix hydrolases than by SpoT, since the expression level of the proteins have not been determined, definitive conclusions cannot be drawn.

When amino acid starvation was induced in a Δ*spoT*/P_*lac*_-spoT^+^ strain after IPTG withdrawal to lower *spoT* expression, only ppGpp accumulation was observed (Figure 4C in Sanyal and Harinarayanan, 2020). As mentioned earlier, absence of pppGpp accumulation after amino acid starvation was also a feature of the *spoT1* strain, which has reduced SpoT hydrolase activity (Laffler and Gallant, 1974). While many strains of *E. coli* accumulate both pppGpp and ppGpp during amino acid starvation. It was noted that strains of the 58–161 lineage (Alfoldi et al., 1962) accumulate ppGpp but not pppGpp during stringent response. This was called the “spotless” phenotype. The genetic locus responsible for the phenotype was called as *spoT* and was defined by a mutant allele that had arisen spontaneously (Laffler and Gallant, 1974). Commonly studied laboratory strains of *E. coli* such as MC4100 also carry the mutant allele of *spoT* (Spira et al., 2008). Based on our results, we believe, the decrease in SpoT hydrolase activity can explain the absence of pppGpp in both instances. However, the molecular basis for the phenotype has not been addressed. Since *mutT* or *nudG* over-expression was able to hydrolyze (p)ppGpp like SpoT (Figure 6), we asked, if the decrease in pppGpp pool associated with reduced SpoT hydrolase activity could be rescued by the expression of the nudix hydrolases.

**FIGURE 7:**
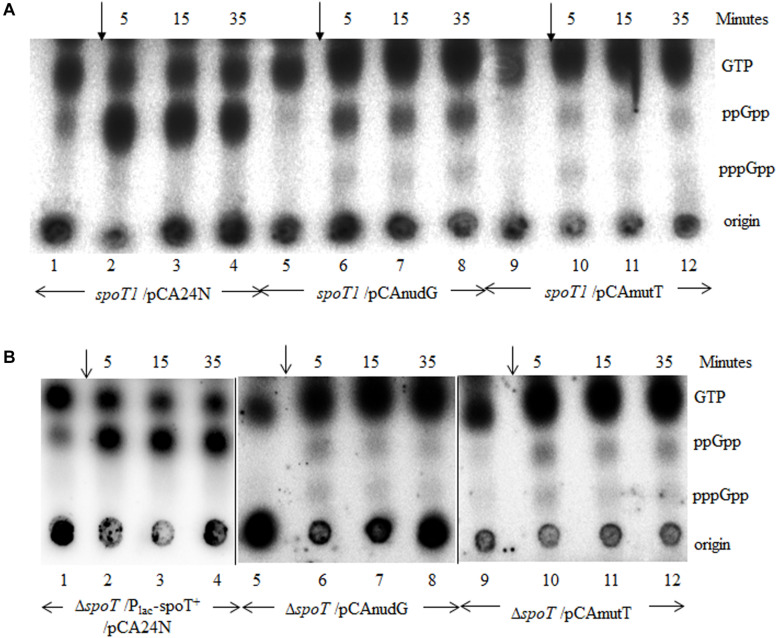
Increased expression of *mutT* or *nudG* lowers the ppGpp pool and elevates pppGpp pool during stringent response in strains with reduced SpoT hydrolase activity. **(A)** Isoleucine starvation was induced by the addition of valine (arrow) to cultures of the *spoT1* strain carrying the vector pCA24N (lanes 1–4), the vector with *nudG* (lanes 5–8), or *mutT* (lanes 9–12). **(B)** Isoleucine starvation was induced in the Δ*spoT*/P_*lac*_-spoT^+^/pCA24N strain after reducing *spoT* + expression by growth in the absence of IPTG (lanes 1–4), in the Δ*spoT*/pCAnudG (lanes 5–8), and Δ*spoT*/pCAmutT (lanes 9–12) strains cultured in the presence of 0.1 mM IPTG. The accumulation of stringent nucleotides was followed with P^32^ labeled cultures as described in the methods. The strains used are, panel **A**, HR1348 (lanes 1–4), HR1350 (lanes 5–8), and HR1349 (lanes 9–12); Panel **B**, RS444 (lanes 1–4), RS460 (lanes 5–8), and RS459 (lanes 9–12).

In the *spoT1* strain carrying the plasmid vector pCA24N, when stringent response was induced by isoleucine starvation, as expected, there was accumulation of ppGpp but not pppGpp (Figure 7A, lanes 1–4). In the *spoT1* strain carrying either pCAnudG or pCAmutT and grown in the presence of IPTG to induce expression of the nudix hydrolases, isoleucine starvation resulted in the accumulation of ppGpp and pppGpp (Figure 7A, lanes 5–12). As compared to the *spoT1*/pCA24N strain, the concentration of ppGpp relative to GTP decreased in the strains induced for *nudG* or *mutT* expression, and this would be expected due to enhanced hydrolysis of ppGpp. The decrease in ppGpp was more pronounced with MutT over-expression than with NudG. This may be attributed to differences in the expression/activity of the two proteins under our experimental conditions or possibly more efficient hydrolysis of ppGpp by MutT than by NudG. Given that pppGpp was not detected during stringent response in the *spoT1*/pCA24N strain, accumulation of this nucleotide in the *spoT1* strain over-expressing a nudix hydrolase indicated a positive correlation between pppGpp level and cellular (p)ppGpp hydrolase activity. Since an increase in hydrolase activity is associated with an increase in pppGpp pool, it would be reasonable to conclude that the absence of pppGpp accumulation in the *spoT1* is not due to hydrolases such as GppA, which specifically target pppGpp.

Since *nudG* or *mutT* over-expression rescued the growth defect of Δ*spoT* strain, we studied the stringent response in Δ*spoT*/pCAnudG and Δ*spoT*/pCAmutT strains and compared it to that seen in the Δ*spoT*/P_*lac*_-spoT^+^/pCA24N strain after *spoT* expression was reduced by IPTG withdrawal. Amino acid starvation after lowering *spoT* expression and without the over-expression of nudix hydrolases resulted in the accumulation of ppGpp but not pppGpp (Figure 7B, lanes 1–4). Amino acid starvation with the over-expression of NudG or MutT lowered the ppGpp pool relative to GTP (Figure 7B, lanes 5–12). This effect of NudG or MutT over-expression was similar to that observed in the wild type or *spoT1* background (Figures 6B,C, 7A) and can be expected from the constitutive degradation of ppGpp to pGp. On the other hand, there was an increase in pppGpp level relative to ppGpp following the expression of nudix hydrolases, once again revealing a positive correlation between pppGpp level and (p)ppGpp hydrolase activity. Since SpoT is also a (p)ppGpp synthase, it may be argued that, reducing the *spoT* expression in Δ*spoT*/P_*lac*_-spoT^+^ strain lowered the pppGpp pool due to reduction in the synthase activity. The recovery of pppGpp pool following the expression of nudix hydrolase would rule out this possibility.

## Discussion

### Physiological Significance of Negative Regulation of RelA-Mediated Stringent Response by SpoT Hydrolase Activity

Because SpoT has a (p)ppGpp hydrolase activity, it could, in theory, negatively regulate the increase in (p)ppGpp pool during RelA mediated stringent response, but verifying this experimentally is important. Firstly, SpoT is a dual function protein with (p)ppGpp synthase (S) and hydrolase activities (H) that are reported to be reciprocally regulated (H^+^ S^–^ or H^–^S^+^) (Hogg et al., 2004) – only the H^+^ S^–^ conformation of the SpoT protein would confer negative regulation. Secondly, it was reported that uncharged tRNA’s inhibit the SpoT hydrolase activity (Richter, 1980), suggesting that an H^–^S^+^ state of SpoT may be possible during amino acid starvation. Thirdly, there are reports with evidence for ppGpp (Shyp et al., 2012) or pppGpp (Kudrin et al., 2018) mediated positive allosteric feedback regulation of RelA *in vitro*, and our earlier study had suggested that an increase in the basal pppGpp pool could activate RelA (Sanyal and Harinarayanan, 2020). Therefore, by regulating the cellular concentration of (p)ppGpp, the SpoT hydrolase activity can modulate the amplification of stringent response. Due to the essential nature of SpoT hydrolase function, experimentally verifying its role in counteracting the RelA-dependent increase in (p)ppGpp is not straightforward. Our experimental evidence for negative regulation by SpoT comes from comparing stringent response in isogenic strains with lowered *relA* expression (Δ*rlmD*:FRT and Δ*rlmD*:FRT Δ*spoT* strains, Figures 2C, 3A). The *rlmD* gene is located immediately upstream of *relA*. Three promoters of *relA* are located within and one at the end of the *rlmD* ORF. Two of these promoters are sigma-54 regulated (Brown et al., 2014) and two by the sigma-70 transcription factor (Metzger et al., 1988; Macia̧g et al., 2011). Therefore, a non-polar deletion of the *rlmD* gene (Δ*rlmD*:FRT) can be expected to reduce the expression of *relA*. The residual expression of *relA* may be supported from the promoter upstream of *rlmD* (Mendoza-Vargas et al., 2009). As there is no evidence in literature to suggest a change in *relA* expression can influence the hydrolase activity of SpoT, the SpoT mediated degradation of ppGpp observed during the stringent response in Δ*rlmD*:FRT strain may be expected in the wild type strain as well. It was reported, uncharged t-RNA’s inhibit the SpoT hydrolase activity (Richter, 1980). This suggests, the SpoT hydrolase activity could be inhibited during amino acid starvation. Further studies are needed to address the biological significance of this finding.

A molecular complex comprising of 70S ribosome with an A-site deacylated tRNA and RelA in a stoichiometry of 1:1:1 initiates stringent response (Cashel et al., 1996). It would be expected that changes in the concentration of RelA or deacylated tRNA will identically affect the concentration of this stringent response ribosomal complex (SRC). After lowering RelA expression, the accumulation of deacylated tRNA elicited stringent response only in the absence of SpoT activity (Figures 2C, 3A). This suggested, a lower concentration of SRC could support (p)ppGpp accumulation in the absence of SpoT hydrolase activity than in a wild type strain. Since SRC concentration would be altered identically from lowering RelA expression or decreasing deacylated tRNA pool, it is plausible that a smaller pool of deacylated tRNA can elicit stringent response in the absence of SpoT hydrolase activity than in the wild type strain. A constitutive SpoT mediated hydrolysis of (p)ppGpp would dampen the amplification of RelA-dependent stringent response, until a quorum of A-site deacylated tRNA molecules are present to cause an aggregate increase in (p)ppGpp pool. It can be argued that SpoT hydrolase activity causes a futile cycling of GTP/GDP to (p)ppGpp to GTP/GDP together with draining of ATP before an increase in (p)ppGpp concentration is apparent. We propose, SpoT hydrolase activity could counteract the onset of stringent response from small contractions in the size of amino acid pool.

In experiments reconstituting RelA-dependent stringent response *in vitro*, it was observed that ppGpp enhanced the rate of RelA-mediated ppGpp synthesis 10-fold in the presence of 70S ribosomes (without starvation signals), leading to the proposal that ppGpp conferred positive allosteric regulation of RelA (Figure 1; Shyp et al., 2012). If such a regulation were to operate *in vivo*, we believe this could be revealed in the Δ*rlmD*:FRT Δ*spoT* strain, where it was possible to maintain substantially high levels of ppGpp without amino acid starvation over prolonged time periods due to the absence of SpoT hydrolase activity (Figure 3A, lanes 5–11). The gradual decrease in ppGpp pool observed here would suggest, if there was any allosteric activation of RelA by ppGpp, it has to be a very weak phenomenon as no increase in ppGpp was detected even in the absence of the primary hydrolase SpoT (which arguably could mask any increase in ppGpp pool from allosteric activation). However, it is possible, the allosteric regulation was not apparent due to reduced expression of *relA* in the strain.

### The Relationship Between pppGpp and SpoT Hydrolase Activity

The over-expression of MutT or NudG lowered the concentration of (p)ppGpp relative to GTP in the wild type strain (Figures 6B,C). This is consistent with the study by Zhang et al. (2018) which showed the over-expression of the proteins lowered the cellular (p)ppGpp pool and hydrolyzed ppGpp to pGp. Although not verified experimentally, pppGpp may also be degraded to pGp. The pGp spot could not be detected under our experimental conditions probably because it co-migrated with GTP. If ppGpp and pppGpp are hydrolyzed with equal efficiency to pGp, then it may be expected that the total amount of this molecule would be similar in the Δ*rlmD*:FRT Δ*spoT* and *spoT1* Δ*gppA*:FRT/P_*lac*_-spoT^+^ strains. This is because, relative to GTP, the ppGpp or (p)ppGpp pool size is similar in the two strains in the absence of amino acid starvation (Supplementary Tables 2, 4). We therefore favor the idea that it was the presence of pppGpp, but not pGp that enhanced the growth inhibition in the latter strain. Reduction in hydrolase activity, as in the *spoT1* strain or the reduced expression of *spoT*, lowers the pppGpp pool relative to ppGpp and GTP during amino acid starvation (Laffler and Gallant, 1974; Sanyal and Harinarayanan, 2020). To our knowledge, the molecular basis for this phenotype has not been addressed. Results from an earlier study suggested the phenotype was not dependent on the pppGpp hydrolase, GppA (Sanyal and Harinarayanan, 2020), but did not definitively rule it out. In the strains deficient for *spoT* hydrolase activity, following the over-expression of nudix hydrolase, the ppGpp concentration dropped relative to GTP as seen in the wild type strain, but interestingly, there was an increase in the pppGpp pool relative to ppGpp (Figure 7). These results suggest, the unexpected decline in pppGpp pool in strains deficient for SpoT hydrolase activity is caused by the decrease in (p)ppGpp hydrolase activity and not the presence of pppGpp hydrolase such as GppA. Further studies are needed to address the molecular basis of the “spotless” phenotype.

## Materials and Methods

### Media and Growth Conditions

LB medium had the final composition of 1% tryptone, 0.5% yeast extract, and 1% NaCl. MOPS buffered minimal medium (Neidhardt et al., 1974), and minimal A medium (Miller, 1992) were prepared as reported. The minimal medium was supplemented with 0.5% glucose, unless mentioned otherwise. In plates, glucose and casamino acids were each supplemented at 0.2% final concentration. serine, methionine, and glycine were supplemented to final concentration of 100 μg ml^–1^ in plates. Antibiotics and their final concentration in the growth medium are ampicillin (Amp) 50 μg ml^–1^, kanamycin (Kan) 25 μg ml^–1^, tetracycline (Tet) 10 μg ml^–1^, and chloramphenicol (Cm) 15 μg ml^–1^. Isopropyl β-D-thiogalactopyranoside (IPTG) was supplemented to final concentration of 1 or 0.1 mM as indicated and 5-Bromo-4-chloro 3-indolyl-β-D-thiogalactoside (X-gal) was used at a final concentration of 50 μg ml^–1^.

### Construction of Strains and Plasmids

All strains were derived from the *E. coli* K-12 strain MG1655. Strains, plasmids, and primers used in this study are tabulated in Supplementary Table 1. Phage P1 mediated transduction was used to introduce mutations into the chromosome following standard protocol (Miller, 1992). Gene deletions were sourced from the Keio collection (Baba et al., 2006), and if necessary, the kanamycin resistance cassette was removed using the FLP recombinase expressed from the pCP20 plasmid (Cherepanov and Wackernagel, 1995). For the construction of *relA496*Δ:Kan and *relA455*Δ:Kan alleles, all codons after 496 or 455 were deleted and replaced with a TAG stop codon and the kanamycin cassette from pKD13 (Datsenko and Wanner, 2000) by recombineering (Yu et al., 2000) using the primers JGOrelA496aaPS4 or JGOrelA455aaPS4 with JGOrelAPS1, respectively. The sequence of the constructs were verified using primers JGOrelA + 882 and K1. The plasmid P_*lac*_-spoT^+^ was constructed using the vector pRC7 (Bernhardt and De Boer, 2004) and has been referred to as pRCspoT previously (Nazir and Harinarayanan, 2016). The *spoT* gene in P_*lac*_-spoT^+^ is under the lac promoter and has the native RBS and the TTG start codon. The plasmids pCA24N, pCAspoT, pCAmutT, and pCAnudG were obtained from the ASKA collection (Kitagawa et al., 2005).

### Depletion of SpoT Using the Plasmid P_*lac*_-spoT^+^

The chromosomal *spoT* gene was either deleted or replaced with *spoT1* in the presence of the single-copy plasmid P_*lac*_-spoT^+^. In this plasmid spoT expression was driven from the *lac* promoter. A tight regulation of expression was expected due to the very low copy number of the plasmid and the plasmid encoding for the LacI repressor protein. Further, there is an elevated expression of *lacI* due to the presence of the *lacI*^*q*^ mutation. Expression of spoT was lowered by the withdrawal of IPTG from the growth medium. Cultures grown in the presence of ampicillin and IPTG (1 mM), were washed with minimal medium to remove IPTG and diluted 100-fold into medium lacking IPTG.

### Plasmid Segregation Assay

The assay works on the principle that an essential gene function provided from an unstable plasmid, would stabilize the plasmid. Since the plasmid carries the *lacZ* gene, blue and white colonies indicate the retention and loss of the plasmid, respectively, in plates containing the inducer IPTG and the indicator X-gal. The assay referred to as the ‘blue–white assay’ and was carried out in strains having the Δ*lacZYAI*:FRT mutation. The absence of white colonies after growth without selection for the plasmid would indicate that the plasmid encoded function was essential under the conditions. Strains carrying P_*lac*_-spoT^+^ were grown in LB broth containing ampicillin and IPTG. Cultures were washed with minimal medium to remove IPTG. For the ‘blue-white’ assay appropriate dilutions of the culture were spread on plates containing IPTG and X-gal so as to obtain ∼300 colonies per plate. Plates were incubated at 37°C for 24 h in the case of LB or minimal A medium containing glucose and casaminoacids and 48 h in the case of minimal A medium with glucose. Incubation was extended to 72 h when white colonies were not evident after 24 or 48 h of incubation. The percentage of white colonies was calculated from the ratio of white colonies over the total number of colonies scored (white + blue).

### (p)ppGpp Estimation by Thin Layer Chromatography

Cultures were grown to saturation in MOPS minimal medium containing 0.5% glucose. These cultures were diluted 100-fold and allowed to grow till an A_600_ of ∼ 0.4 to 0.5, and then diluted 10-fold into a pre-warmed low-phosphate medium with 0.4 mM K_2_HPO_4_ and 100–200 μCi ml^–1^ of ^32^P-H_3_PO_4_. After at least two doublings in this medium, isoleucine starvation was induced by the addition of valine. An unlabeled culture was used to monitor A_600_. Samples were collected in tubes containing an equal volume of 2 N HCOOH kept chilled on ice, subjected to 3 cycles of freeze-thaw and centrifuged at 10000 rpm for 5 min at 4°C. The supernatant was spotted on PEI cellulose sheets and resolved using 1.5 M KH_2_PO_4_, pH 3.4. The nucleotide spots were imaged using phosphorimager (Typhoon FLA 9500). Quantification of the spots was carried out by densitometry after subtracting background, using the multi-gage V3.0 software (Fujifilm).

### Isolation of Multi-Copy Suppressors

The Δ*rlmD*:FRT Δ*spoT* Δ*gppA*/P_*lac*_-spoT^+^ strain was transformed with an *E. coli* genomic library made in plasmid pACYC184 (Saxena and Gowrishankar, 2011) and chloramphenicol resistant transformants were selected on media without ampicillin and IPTG which is non-permissive for the parental strain. Minimal A glucose medium with or without casaminoacids was used to select the plasmid clones that rescued the growth defect of the parental strain (from approximately 80,000 transformants obtained in each medium). A total of 72 ampicillin sensitive white colonies were identified, which indicated that the growth of these clones were independent of SpoT function. When plasmid isolated from 25 out of the 72 colonies was individually transformed into the Δ*rlmD*:FRT Δ*spoT* Δ*gppA*/P_*lac*_-spoT^+^ strain, 22 were found to support growth of Δ*rlmD*:FRT Δ*spoT* Δ*gppA* strain. Four out of the 22 plasmids also rescued the growth defect of the Δ*relA* Δ*spoT* (ppGpp^0^) strain in minimal A glucose medium, and therefore, thought to carry the *spoT* gene and sequencing one of the 4 clones showed this was indeed the case. After sequencing the other 18 clones, unique DNA fragments were identified in five clones.

### Isoleucine Starvation and Reversal of Starvation

The addition of valine inhibits isoleucine biosynthesis in *E. coli* K-12 strains causing isoleucine starvation. This is because, enzymes catalyzing the first common step in the biosynthesis of the branched chain amino acids isoleucine and valine are feedback inhibited by valine and the feedback resistant enzyme encoded by the *ilvG* gene is inactive in this strain due to a chain terminating mutation (Umbarger and Brown, 1957). Valine was added to a final concentration of 100 μg ml^–1^ to induce isoleucine starvation, and this was reversed by the addition of 100 μg ml^–1^ of isoleucine.

## Data Availability Statement

All datasets presented in this study are included in the article/Supplementary Material.

## Author Contributions

RH conceived the study. RS and RH designed the experiments. RS and AV performed the experiments. All authors contributed to the article and approved the submitted version.

## Conflict of Interest

The authors declare that the research was conducted in the absence of any commercial or financial relationships that could be construed as a potential conflict of interest.
