# Supplementary material for: Studies on the Regulation of (p)ppGpp Metabolism and Its Perturbation Through the Over-Expression of Nudix Hydrolases in Escherichia coli
Source: Front Microbiol. 2020 Oct 15;11:562804. doi: 10.3389/fmicb.2020.562804 (PMC7593582; doi:10.3389/fmicb.2020.562804)
Supplement: Supplementary file 1 [file Data_Sheet_1.PDF]

Supplementary Material

1 Supplementary Tables

Table S1. List of strains, plasmids, and primers

Strain Number	Relevant genotype or features	Source or reference
MG1655	*F− λ− ilvG– rfb-50 rph-1* (Wild-type *E. coli* K-12)	Lab collection
BW25113	Δ*(araB–D)567* Δ*(rhaD–B)568* Δ*lacZ4787*(::*rrnB-3*) *hsdR514 rph-1*	(Baba et al., 2006)
CF1693	CF1648 *relA251*::Kan *spoT207*::Cm	(strain collection, Cashel Lab)
CF10237	CF1648 Δ*relA* (*relA256*) Δ*spoT* (*spoT212*)	(strain collection, Cashel Lab)
JW2755	BW25113 Δ*relA*::Kan	(Baba et al., 2006)
JW5603	BW25113 Δ*gppA::*Kan	(Baba et al., 2006)
JW2756	BW25113 Δ*rlmD*::Kan	(Baba et al., 2006)
AN131	Δ*lacZYAI*::FRT/ P_{lac}-spoT^{+}	(Nazir and Harinarayanan, 2016)
RS1	MG1655 Δ*lacZYAI*::FRT	(Nazir and Harinarayanan, 2016)
RS8	Δ*lacZYAI*::FRT Δ*relA::*FRT	This work
RS9	Δ*lac relA496*::Tn*10*dTet	This work
RS11	Δ*lac rlmD*::Tn*10*dKan	This work

RS17	Δ*lacZYAI*::FRT *spoT207*::Cm r*elA496*::Tn1*0*dTet/P_{lac}-spoT$^+$	This work
RS18	Δ*lacZYAI*::FRT *spoT207*::Cm *rlmD*::Tn*10*dKan/ P_{lac}-spoT$^+$	This work
RS24	Δ*lacZYAI*::FRT *spoT1*	This work
RS39	Δ*lacZYAI*::FRT *relA1*	This work
RS53	Δ*lacZYAI*::FRT *relA455*Δ::Kan	This work
RS54	Δ*lacZYAI*::FRT *relA496*Δ::Kan	This work
RS92	Δ*lacZYAI*::FRT Δ*spoT relA455*Δ::Kan/ P_{lac}-spoT$^+$	This work
RS194	Δ*lacZYAI*::FRT *spoT1* Δ*gppA*::FRT/ P_{lac}-spoT$^+$	This work
RS303	Δ*lac* Δ*rlmD*::Kan	This work
RS307	Δ*lacZYAI*::FRT Δ*gpp*A::FRT/ P_{lac}-spoT$^+$	This work
RS316	Δ*lac* Δ*rlmD*::FRT	This work
RS361	Δ*lacZYAI*::FRT Δ*spoT* Δ*rlmD*::FRT/ P_{lac}-spoT$^+$	This work
RS415	Δ*lacZYAI*::FRT Δ*rlmD*::FRT Δ*spoT* Δ*gppA::*FRT/ P_{lac}-spoT$^+$	This work
RS420	Δ*lacZYAI*::FRT *relA496*Δ::Kan Δ*spoT*/ P_{lac}-spoT$^+$	This work
RS443	Δ*lacZYAI*::FRT Δ*rlmD*::FRT Δ*spoT* Δ*gppA*::FRT/ P_{lac}-spoT$^+$/ pACYC184	This work
RS444	Δ*lacZYAI*::FRT Δ*spoT*/Plac-spoT+ /pCA24N	This work
RS459	Δ*lacZYAI*::FRT Δ*spoT*/pCAmutT	This work
RS460	Δ*lacZYAI*::FRT Δ*spoT*/pCAnudG	This work

RS650	Δ*lacZYAI*::FRT Δ*rlmD*::FRT Δ*spoT* Δ*gppA*::FRT/ P_{lac}-spoT^{+}/ pACYC- *rpoZ'-spoT-trmH-recG'*	This work
RS651	Δ*lacZYAI*::FRT Δ*rlmD*::FRT Δ*spoT* Δ*gppA*::FRT/ P_{lac}-spoT^{+}/ pACYC- *ynjC'-ynjD-ynjE-ynjF-nudG-ynjH-gdhA'*	This work
RS654	Δ*lacZYAI*::FRT Δ*rlmD*::FRT Δ*spoT* Δ*gppA*::FRT/ P_{lac}-spoT^{+}/ pACYC- *rhlB'-gppA-rep'*	This work
RS656	Δ*lacZYAI*::FRT Δ*rlmD*::FRT Δ*spoT* Δ*gppA*::FRT/ P_{lac}-spoT^{+}/ pACYC- *nudG-ynjF-ynjE-ynjD-ynjC'*	This work
RS658	Δ*lacZYAI*::FRT Δ*rlmD*::FRT Δ*spoT* Δ*gppA*::FRT/ P_{lac}-spoT^{+}/pACYC-*coaE-zapD-yacG-mutT-secA*	This work
RS659	Δ*lacZYAI*::FRT Δ*rlmD*::FRT Δ*spoT* Δ*gppA*::FRT/ P_{lac}-spoT^{+}/pACYC-*ynjH-nudG-ynjF*	This work
RS680	Δ*lacZYAI*::FRT Δ*spoT*/ P_{lac}-spoT^{+}/pCAmutT	This work
RS681	Δ*lacZYAI*::FRT Δ*spoT*/ P_{lac}-spoT^{+}/pCAnudG	This work
RS684	Δ*lacZYAI*::FRT Δ*spoT* Δ*gppA*::Kan/ P_{lac}-spoT^{+}/pCA24N	This work
RS685	Δ*lacZYAI*::FRT Δ*spoT* Δ*gppA*::Kan/ P_{lac}-spoT^{+}/pCAmutT	This work
RS686	Δ*lacZYAI*::FRT Δ*spoT* Δ*gppA*::Kan/ P_{lac}-spoT^{+}/pCAnudG	This work
RS688	Δ*lacZYAI*::FRT/pCA24N	This work
RS689	Δ*lacZYAI*::FRT/pCAmutT	This work
RS690	Δ*lacZYAI*::FRT/pCAnudG	This work
RS760	Δ*lacZYAI*::FRT/pCAspoT	This work
HR1348	Δ*lacZYAI*::FRT *spoT1*/ pCA24N	This work
HR1349	Δ*lacZYAI*::FRT *spoT1*/ pCAmutT	This work
HR1350	Δ*lacZYAI*::FRT *spoT1*/ pCAnudG	This work

Plasmids	Description	Source
pCP20	A pSC101-based temperature sensitive plasmid used for *in vivo* expression of Flp recombinase.	(Cherepanov and Wackernagel, 1995)
pKG137	A modified version of pCE37 having an optimal RBS ahead of *lacZ.*	(Ellermeier et al., 2002)
P_{lac}-spoT+	pRC7 which contains a minimal *spoT* ORF PCR amplified from the MG1655 and ligated in its *Eco*RI and *Hin*dIII sites, the expression of which is under *lac* promoter, therefore IPTG-inducible	(Nazir and Harinarayanan, 2016)
pCA24N	Vector backbone for ASKA clones	(Kitagawa et al., 2005)
pCAmutT	JW0097-ASKA Collection	(Kitagawa et al., 2005)
pCAnudG	JW1748-ASKA Collection	(Kitagawa et al., 2005)
pCAspoT	JW3625-ASKA Collection	(Kitagawa et al., 2005)
**Primers**	**Oligonucleotide sequence**	
JGOrelA496aaPS4	CACAACCAGCCGTGGGCGTTCGAAAATTCACGCCTGGTTCTAGATTCCGGGGATCCGTCGACC	
JGOrelA455aaPS4	AAATTGGCGGGCGCATTGTGCCGTTCACCTACCAGCTGCAGTAGATTCCGGGGATCCGTCGACC	
JGOrelAPS1	TACAGTATATATCAATCTACATTGTAGATACGAGCAAATTTCGGCGTGTAGGCTGGAGCTGCTTC	
JGOrelA+882	CATCTACAGCATCTGGCG	

Table S2. The ratio of GTP or ppGpp to total (ppGpp + GTP) in the Δ*rlmD*::FRT Δ*spoT* strain following isoleucine starvation and reversal of starvation. Data obtained from three independent experiments.

**Δ*rlmD*::FRT Δ*spoT***	**Nucleotide/total**	
	**GTP/(GTP+ppGpp)**	**ppGpp/(GTP+ppGpp)**
Basal level	0.883 ± 0.025	0.117 ± 0.025
Val-5 min	0.617 ± 0.032	0.383 ± 0.032
Val-15 min	0.491 ± 0.027	0.509 ± 0.027
Val-35 min	0.496 ± 0.022	0.504 ± 0.022
Ile-1 min	0.498 ± 0.035	0.502 ± 0.035
Ile-30 min	0.538 ± 0.025	0.462 ± 0.025
Ile-60 min	0.571 ± 0.033	0.429 ± 0.033
Ile-90 min	0.617 ± 0.037	0.383 ± 0.037
Ile-120 min	0.645 ± 0.057	0.355 ± 0.032
Ile-150 min	0.670 ± 0.046	0.330 ± 0.046
Ile-180 min	0.692 ± 0.056	0.308 ± 0.042

Table S3. The ratio of GTP or ppGpp to total (GTP+ppGpp) in the *spoT1* mutant following isoleucine starvation and reversal of starvation. Data obtained from three independent experiments.

***spoT1* strain**	**Nucleotide / total**	
	**GTP/total**	**ppGpp/total**
Basal Level	0.879 ± 0.032	0.121 ± 0.032
Val – 15 min	0.290 ± 0.012	0.710 ± 0.012
Ile – 1min	0.325 ± 0.021	0.675 ± 0.021
Ile – 5 min	0.405 ± 0.012	0.595 ± 0.012
Ile – 15 min	0.636 ± 0.021	0.364 ± 0.021
Ile – 20 min	0.765 ± 0.034	0.235 ± 0.034

Table S4. The ratio of GTP or ppGpp or pppGpp to total (GTP+ppGpp+pppGpp) in the *spoT1* Δ*gppA*/P_{lac}-spoT+ strain after SpoT depletion and followed by isoleucine starvation and reversal of starvation. Data obtained from three independent experiments.

***spoT1* Δ*gppA*/ P_{lac}-spoT+**	**Nucleotide / total**		
	**GTP/total**	**ppGpp/total**	**pppGpp/total**
Basal Level	0.658 ± 0.04	0.254 ± 0.015	0.088 ± 0.025
Val – 35 min	0.382 ± 0.035	0.421 ± 0.007	0.198 ± 0.029
Ile – 1 min	0.398 ± 0.034	0.419 ± 0.01	0.183 ± 0.027
Ile – 5 min	0.441 ± 0.033	0.396 ± 0.012	0.163 ± 0.026
Ile – 15 min	0.515 ± 0.037	0.335 ± 0.015	0.150 ± 0.026
Ile – 35 min	0.623 ± 0.037	0.262 ± 0.018	0.115 ± 0.021

Table S5. The ratio of GTP or ppGpp or pppGpp to total (GTP+ppGpp+pppGpp) in the WT and Δ*gppA* mutant following isoleucine starvation and reversal of starvation. Data obtained from two independent experiments.

	**Strain**	**Nucleotide / total**		
		**GTP / total**	**ppGpp / total**	**pppGpp / total**
Basal Level	WT	0.917 ± 0.019	0.073 ± 0.015	0.010 ± 0.003
	Δ*gppA*	0.904 ± 0.024	0.048 ±0.010	0.048 ± 0.014
Val – 5 min	WT	0.421±0.039	0.411±0.020	0.168±0.019
	Δ*gppA*	0.181±0.017	0.313±0.018	0.506±0.011
Val – 15 min	WT	0.439±0.041	0.398±0.016	0.163±0.026
	Δ*gppA*	0.191±0.009	0.311±0.012	0.498±0.018
Val – 35 min	WT	0.467 ± 0.049	0.378 ± 0.019	0.155 ± 0.030
	Δ*gppA*	0.197 ± 0.004	0.308 ± 0.010	0.495 ± 0.014
Ile – 1 min	WT	0.979 ± 0.013	0.006 ± 0.005	0.015 ± 0.007
	Δ*gppA*	0.728 ± 0.052	0.167 ± 0.033	0.105 ± 0.019
Ile – 2 min	WT	0.968 ± 0.025	0.011 ± 0.001	0.021 ± 0.024
	Δ*gppA*	0.932 ± 0.001	0.032 ± 0.013	0.036 ± 0.012

Table S6. Suppression of Δ*rlmD* Δ*spoT* Δ*gppA* synthetic lethality and growth defect of Δ*relA* Δ*spoT* strain by the multi-copy suppressors.

**Chromosomal DNA in the multi-copy clones (strain number)**	**Suppression of Δ*rlmD* Δ*spoT* Δ*gppA* growth defect on LB IPTG X-gal**	**Growth of Δ*relA* Δ*spoT* on minimal glucose**
pACYC184 (vector) (RS443)	<0.5% (<1/196)[a]	–
rpoZ'-spoT-trmH-recG' (RS650)	42% (72/172)	+
rhlB'-gppA-rep' (RS654)	64% (56/157)	–
ynjC'-ynjD-ynjE-ynjF-nudG-ynjH-gdhA' (RS651)	23% (42/180)	–
ynjH'-nudG-ynjF' (RS659)	43% (146/340)	–
nudG-ynjF-ynjE-ynjD-ynjC' (RS656)	59% (93/157)	–
coaE'-zapD-yacZ-mutT-secA' (RS658)	18% (51/280)	–

a - The percentage of white colonies and the number of white colonies over the total number of colonies scored (white+blue).

Table S7. Over-expression of *mutT* and *nudG* and SMG resistance phenotype of Δ*relA spoT202* and Δ*relA spoT203* strains.

**Genotype**	**minimal medium[a]**	
	**None**	**SMG (100 µg ml^{-1})**
Δ*relA spoT202* /pCA24N	++[b]	+
Δ*relA spoT202* /pCAmutT	+++	−
Δ*relA spoT202* /pCAnudG	+++	−
Δ*relA spoT203* /pCA24N	+	+
Δ*relA spoT203* /pCAmutT	+++	−
Δ*relA spoT203* /pCAnudG	+++	−

a – contains Cm and 0.1 mM IPTG. b – Growth at 37°C after 24 hours for minimal medium and up to 72 hours for plates containing SMG. b - +++, ++, + and − indicate decreasing size of colonies and no growth.

2 Supplementary Figures

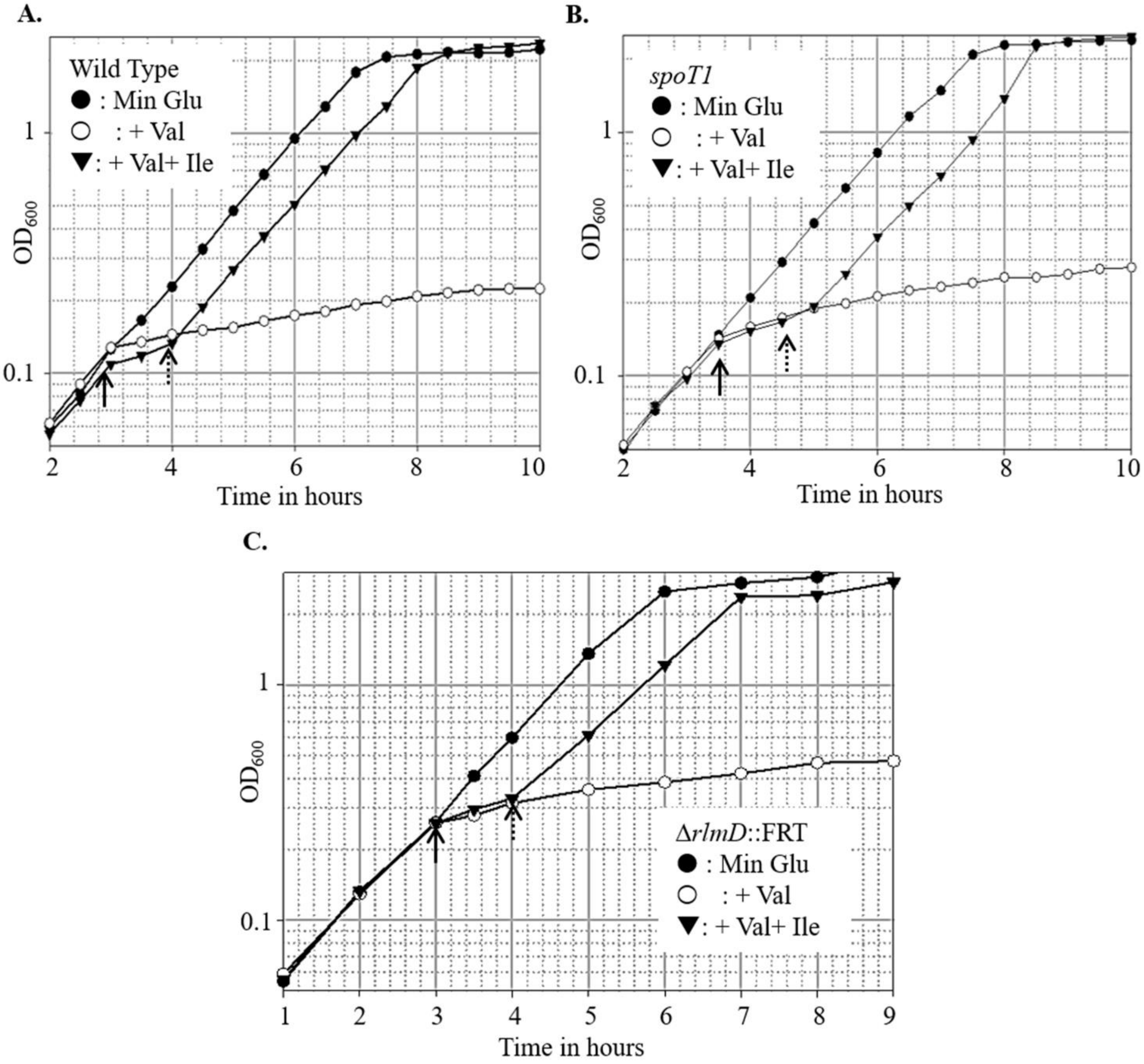

Figure S1. Growth of wild type (A), *spoT1* (B) and Δ*rlmD* (C) strains in MOPS minimal medium containing glucose or after valine addition (arrow) or after valine addition and followed by isoleucine addition (dotted arrow). Strains are RS1, RS24, and RS316.

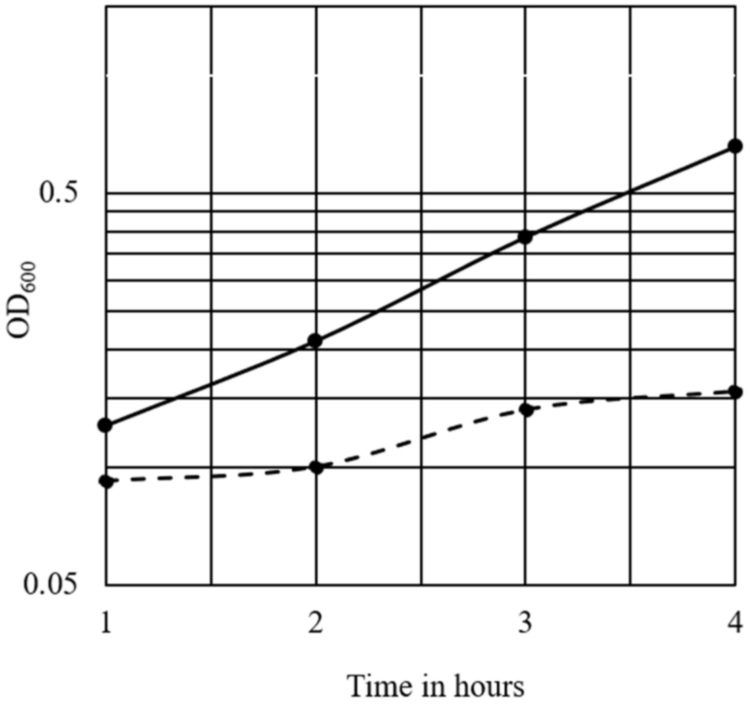

Figure S2. Growth inhibition associated with accumulation of (p)ppGpp. Growth of the *spoT1* Δ*gppA*::FRT/P_{lac}-spoT$^+$ (RS194) strain was followed in minimal medium containing ampicillin either in the presence of 1 mM IPTG to induce *spoT* expression (solid line) or in the absence of IPTG (dashed line) to lower *spoT* expression.

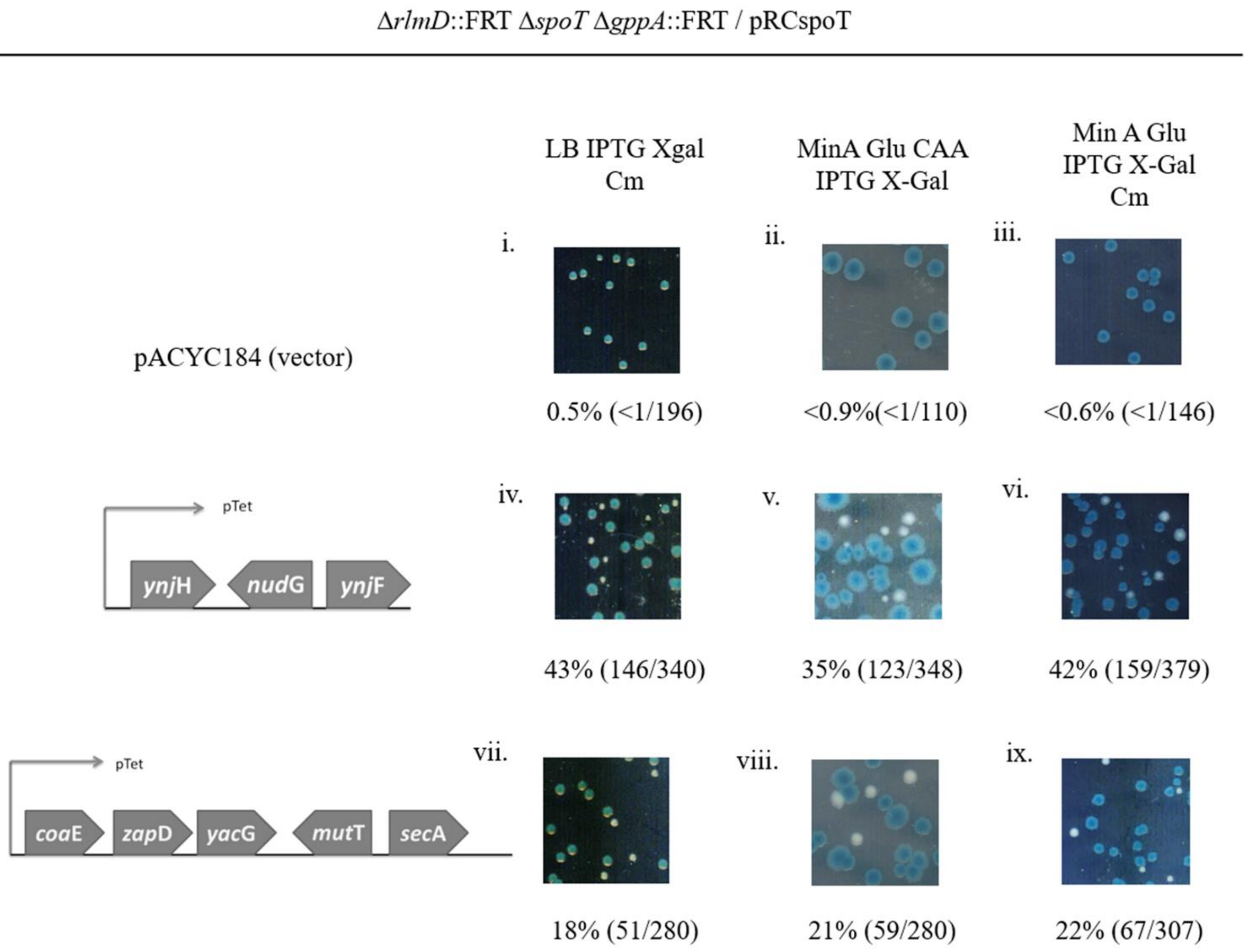

Figure S3. Suppression of Δ*rlmD* Δ*spoT* Δ*gppA* synthetic lethality by two representative genomic DNA fragments carried on plasmid pACYC184. Plasmid segregation assay was used to monitor plasmid loss or retention in the growth media indicated. Representative section from the plates have been included for each strain to show the color of the colonies after non-selective growth. The percentage of white colonies and the number of white colonies over the total number of colonies scored (white + blue) is provided for each panel. A cartoon of chromosomal DNA fragment with genes (full length or truncated) has been shown. The strains used are RS443 (panels i to iii), RS659 (panels iv to vi) and RS658 (panels vii to ix). The genes *ynjH*, *ynjF*, *coaE*, and *secA* are truncated.

3 References

Baba, T., Ara, T., Hasegawa, M., Takai, Y., Okumura, Y., Baba, M., et al. (2006). Construction of *Escherichia coli* K-12 in-frame, single-gene knockout mutants: the Keio collection. Mol. Syst. Biol. 2, 2006.0008.

Cherepanov, P. P., and Wackernagel, W. (1995). Gene disruption in *Escherichia coli*: TcR and KmR cassettes with the option of Flp-catalyzed excision of the antibiotic-resistance determinant. Gene. 158, 9–14.

Ellermeier, C. D., Janakiraman, A., and Slauch, J. M. (2002). Construction of targeted single copy lac fusions using λ Red and FLP-mediated site-specific recombination in bacteria. Gene. 290, 153–161.

Kitagawa, M., Ara, T., Arifuzzaman, M., Ioka-Nakamichi, T., Inamoto, E., Toyonaga, H., et al. (2005). Complete set of ORF clones of *Escherichia coli* ASKA library (A complete set of *E. coli* K-12 ORF archive): unique resources for biological research. DNA Res. 12, 291–299.

Nazir, A., and Harinarayanan, R. (2016). Inactivation of cell division protein FtsZ by SulA makes Lon indispensable for the viability of a $ppGpp^0$ strain of *Escherichia coli*. J. Bacteriol. 198, 688–700.
